# Folic Acid Mitigates Angiotensin-II-Induced Blood Pressure and Renal Remodeling

**DOI:** 10.1371/journal.pone.0083813

**Published:** 2013-12-26

**Authors:** Sathnur B. Pushpakumar, Sourav Kundu, Naira Metreveli, Utpal Sen

**Affiliations:** Department of Physiology and Biophysics, University of Louisville School of Medicine, Louisville, Kentucky, United States of America; Cedars-Sinai Medical Center, United States of America

## Abstract

Clinical data suggests an association between systolic hypertension, renal function and hyperhomocysteinemia (HHcy). HHcy is a state of elevated plasma homocysteine (Hcy) levels and is known to cause vascular complications. In this study, we tested the hypothesis whether Ang II-induced hypertension increases plasma Hcy levels and contributes to renovascular remodeling. We also tested whether folic acid (FA) treatment reduces plasma Hcy levels by enhancing Hcy remethylation and thus mitigating renal remodeling. Hypertension was induced in WT mice by infusing Ang II using Alzet mini osmotic pumps. Blood pressure, Hcy level, renal vascular density, oxidative stress, inflammation and fibrosis markers, and angiogenic- and anti-angiogenic factors were measured. Ang II hypertension increased plasma Hcy levels and reduced renal cortical blood flow and microvascular density. Elevated Hcy in Ang II hypertension was associated with decreased 4, 5-Diaminofluorescein (DAF-2DA) staining suggesting impaired endothelial function. Increased expression of Nox-2, -4 and dihydroethidium stain revealed oxidative stress. Excess collagen IV deposition in the peri-glomerular area and increased MMP-2, and -9 expression and activity indicated renal remodeling. The mRNA and protein expression of asymmetric dimethylarginine (ADMA) was increased and eNOS protein was decreased suggesting the involvement of this pathway in Hcy mediated hypertension. Decreased expressions of VEGF and increased anti-angiogenic factors, angiostatin and endostatin indicated impaired vasculogenesis. FA treatment partially reduced hypertension by mitigating HHcy in Ang II-treated animals and alleviated pro-inflammatory, pro-fibrotic and anti-angiogenic factors. These results suggest that renovascular remodeling in Ang II-induced hypertension is, in part, due to HHcy.

## Introduction

Renovascular injury and fibrosis due to angiotensin II (Ang II) is a leading cause of cardio-renovascular morbidity and mortality. Clinical data suggest an association between elevated levels of homocysteine (Hcy), known as hyperhomocysteinemia (HHcy), and systolic hypertension [1]. In addition, plasma Hcy level has an inverse relation with renal function [2]. Although Ang II has predominant actions on the renal vasculature causing a reduction in renal blood flow, the effect of HHcy and its contribution to renovascular remodeling in Ang II-induced hypertension is unclear.

HHcy induces reactive oxygen species (ROS) production by auto-oxidation or by homocysteinylation of lysine residues of other cellular proteins [3]. In addition, HHcy is also known to decrease the antioxidant status [4]. The generation of ROS triggers leukocyte infiltration and cytokine release leading to glomerular inflammation and subsequent injury [5], [6]. Chronic HHcy has also been reported to alter ECM components contributing to glomerulosclerosis [7], [8]. Matrix metalloproteinases (MMPs) and their endogenous inhibitors, tissue inhibitors of metalloproteinases (TIMPs), play a major role in ECM remodeling under physiological and pathological conditions [9], [10]. Although the kidney expresses all the currently described TIMPs, (TIMP-1 - 4) their expression and activities are varied [11]–[13]. TIMP-1, -2 and -4 mediate their action by blocking the MMPs' catalytic core, whereas TIMP-3 binds to ECM and protects it from MMP mediated injury [14]. Thus, TIMPs regulate ECM by inhibiting MMPs. HHcy induces MMP-2, -9 [13] and also modulates TIMPs [15] to promote matrix accumulation [16]; however, whether a similar mechanism is involved in Ang II-induced kidney remodeling has not been reported.

During vascular remodeling, vascular endothelial growth factor (VEGF) plays an important role by promoting endothelial cell proliferation, migration and tube formation [17]. However, during HHcy these processes are inhibited suggesting impairment of vessel growth [18], [19]. Additionally, HHcy induced MMP activation can also lead to increased production of anti-angiogenic factors, endostatin and angiostatin, further inhibiting vascular growth by down regulation of VEGF [20]. The anti-angiogenic molecules specifically target endothelial cells to inhibit proliferation, survival, migration, and sprouting [21]. Since VEGF is widely expressed in the kidney, the consequences of VEGF inhibition can result in loss of vascular and glomerular integrity leading to renal dysfunction [22], [23].

Folic acid (FA) is a B-vitamin which acts as a co-factor in the Hcy remethylation pathway to reduce plasma Hcy level and thus reducing Hcy-induced oxidative stress and DNA damage [24]. However, the role of FA in hypertension-induced HHcy, glomerular injury, inflammation, and subsequent glomerulosclerosis remains largely unknown. The current study was undertaken to delineate the potential role of Hcy in Ang II-induced hypertension and renovascular remodeling. Additionally, considering its potential effects to reduce Hcy levels, FA was given to mitigate Hcy mediated renal damage.

## Materials and Methods

### Animal groups and protocol

Wild type (WT, C57BL/6J) mice were obtained from Jackson Laboratories (Bar Harbor, ME) and housed in the animal care facility at University of Louisville. All animal procedures were performed in accordance with the National Institute of Health Guidelines for animal research and were approved by the Institutional Animal Care and Use Committee of the University of Louisville, School of Medicine.

Animals were allocated into the following groups: 1) Vehicle (saline), 2) Ang II, 3) Ang II + FA, and 4) FA. Hypertension was created by infusing Ang II (1000 ng/kg/min) using Alzet mini osmotic pump intraperitoneally for 4 weeks. Folic acid was given at 0.015 g/L in drinking water starting from 2 weeks after Ang II pump insertion and continued till the end of the experiment. Water was changed on alternate days. In previous studies, FA has been given by different routes and at varied concentration from 0.1425 to 375 µg/25 g b.w./day [25], [26]. Since FA in drinking water changes the taste, we chose a dose based on previous work from our laboratory [27] and also to ensure adequate water consumption.

### Antibodies and chemicals

Primary antibodies against angiostatin, endostatin, MMP-2, MMP-9, and Nox2 were procured from Abcam Inc. (Cambridge, MA). Nox4 was purchased from EMD Millipore Corporation (Billerica, MA). Anti-mouse VEGF antibody was from R&D Systems (Minneapolis, MN). The TIMP-2, TIMP4 and HRP-linked secondary antibodies were from Santa Cruz Biotechnology (Santa Cruz, CA). The β-actin antibody, anti-collagen IV antibody, Angiotensin II, folic acid, 4, 5-Diaminofluorescein (DAF-2DA) and other analytical reagents were from Sigma-Aldrich (St. Louis, MO). Dihydroethidium (DHE) was purchased from Molecular Probes (NY), Barium sulfate was purchased from Acros Organics (part of Thermo Fisher Scientific). PVDF membrane was from Bio-Rad (Hercules, CA).

### DSI radiotelemetry

Blood pressure was measured in conscious mice using DSI radio telemetric system (Data Sciences International; St. Paul, MN). Briefly, a pressure transducer (PA-C20) was surgically implanted into the aortic arch through the left common carotid artery. Animals were allowed to recover for one week before measuring blood pressure. Data was collected and analyzed with DSI Dataquest ART 3.1 software.

### High Performance Liquid Chromatography (HPLC)

The measurement of plasma Hcy has been described in detail previously [28]. Briefly, in a 2 ml tube the following were added: 200 µl of plasma, 100 µl of water, 300 µl of 9M urea (pH 9.0), 50 µl of antifoaming agent n-amyl alcohol, and 50 µl 10% NaBH_4_ solution (wt/vol in 0.1N NaOH). The reaction was conducted in 50°C incubation bath for 30 min. Sample was cooled down to root temperature and 500 µl of 20% trichloroacetic acid was added to precipitate protein. Sample was then centrifuged at 12,000 g for 5 min, and supernatant was filtered through 0.22 µm filter. The filtered supernatant was the final sample for HPLC analysis. The mobile phase solution was a mixture of 0.1 M monochloroacetic acid and 1.8 mM octylsulfate in HPLC grade water, at pH 3.2. The constant flow rate of isocratic solvent was 0.8 ml/min. A Shimadzu Class-VP 5.0 chromatograph (Shimadzu) system was used to analyze samples.

### Laser Doppler study

Renal cortical blood flow was measured using Speckle Contrast Imager (MoorFLPI, Wilmington, DE). The camera was positioned approximately 15 cms from the dorsal surface of left kidney; settings for low-resolution/high-speed images included a display rate of 25 Hz, time constant of 1.0 s, and camera exposure time of 20 ms. The contrast images were processed to produce a flux units trace and recorded for 2 min. To minimize the effect of anesthesia and temperature on BP and blood flow to the kidney, we used the same dose of 2, 2, 2 tribromoethanol (TBE) and maintained standard conditions during measurements.

### Barium X-ray angiography

Renal angiogram was performed by infusing 1 ml Barium sulfate solution (100 mg/mL at pH 5.0) at a constant rate (200 µL/mL) through the carotid artery into the aorta using a PE-10 catheter (ID-0.28 mm, BD Intramedic, Franklin Lakes, NJ). The whole kidney was imaged in a KODAK 4000MM Digital Imaging Station.

### DAF-2DA staining for nitric oxide (NO)

Kidney sections of 5 µm thickness were washed with Hanks balanced salt solution (HBSS). DAF-2DA fluorescent probe (10 µM) was applied for 1 h in dark at 37°C. In order to stimulate NO release, the sections were exposed to acetylcholine at 10^−5^ concentration, 15 minutes after the application of DAF-2DA. After washing with PBS, nuclei were counterstained with 4′,6-diamidino-2-phenylindole (DAPI) at 300 nM concentration for 5 min. Sections were washed twice with PBS and mounted using Fluoroshield containing DABCO antifading media (Sigma Aldrich, St. Louis, MO). Images were captured on an Olympus microscope (B&B Microscope, Pittsburgh, PA). Upto 10 independent fields in 3–5 confocal images of intrarenal arteries were examined in each group and analyzed using Image-Pro-Plus software (Media Cybernetics, Rockville MD).

### Detection of reactive oxygen species (ROS)

Dihydroethidium (DHE; Invitrogen, Carlsbad, CA), was used to detect ROS in frozen kidney sections. Kidney sections, 5 μm thick, were fixed in ice cold acetone for 10 min and air dried. Sections were incubated with DHE (5 µM/L) in a dark humidified chamber for 10 min at room temperature. After washing with PBS, slides were mounted with FluoroGel mounting medium (GeneTex Inc., Irvine, CA). Images were taken by laser scanning confocal microscope (Olympus FluoView 1000, Pittsburgh, PA) and analyzed as above.

### Histology

Kidney samples were fixed in 3.7% formaldehyde and processed to make 5 µm thick sections. Masson's trichrome staining (Thermo Fisher Scientific, Hudson, NH) was done to detect collagen following manufacturers' instructions. Collagen appears as blue color.

### Reverse transcription polymerase chain reaction

Snap frozen kidney samples were processed for RNA extraction using the TRIzol isolation method according to the manufacturer's protocol. The quality of total RNA was determined by NanoDrop ND-1000 and only highly pure quality RNAs (260/280- 2.00 and 260/230-2.00) were used for semi quantitative PCR. All the primers were purchased from Invitrogen (Carlsbad, CA) and Table 1 shows the detail list.

**Table 1:** Forward and reverse primer sequence used.

mRNA	Orientation	Primers (5′-3′)
CBS	Forward	TGCGGAACTACATGTCCAAG
	Reverse	TTGCAGACTTCGTCTGATGG
CSE	Forward	GACCTCAATAGTCGGCTTCGTT
	Reverse	CAGTTCTGCGTATGCTCCGTAA
MTHFR	Forward	TACCAGCCCTATAATACCCC
	Reverse	AAATAGTCAGCAAACTCGGT
ADMA	Forward	AAATGCAACTTTGGATGGTG
	Reverse	AAGAGTTAGTAGCACAGTGG
GAPDH	Forward	TAAATTTAGCCCGTGTGACCT
	Reverse	AGGGGAAAGACTGAGAAAAC

Semi-Quantitative Reverse Transcription-PCR (RT-PCR): The total RNA (400–500 ng) was reverse transcribed by two-step process using ImProm-II™ RT-PCR kit (Promega Corp. Madison, WI). Incubation of RNA with oligodT at 70°C for 5.00 min was performed in thermocycler (Bio-Rad). After quick chill, RT enzyme was added to each samples and the RT cycle was set to 25°C for 2.00 min, 42°C for 50.00 min, 75°C for 5.00 min, and 4°C at the end. After RT, the products were used for amplification. PCR program for amplification of cDNA was set to 95°C for 10.00 min, (95°C for 00.30 min, 58°C for 1.00 min, 72°C for 00.30 min) x 35 cycles, 95°C for 1.00 min, 55°C for 00.30 min and 95°C for 00.30 min. Following amplification, the samples were loaded and run in a 2% agarose gel. The gel was analyzed by Bio-Rad image lab analyzer and software.

### Western blotting analysis

Western blot analysis was performed according to the following protocol. Equal amount of protein was separated by SDS-PAGE, transferred to PVDF membrane and blocked with 5% non-fat milk in TBS solution (W/V). Membrane was washed in TBS-T (TBS + Tween 20) thrice and incubated with appropriate primary antibody overnight. After washing, membrane was incubated for an hour with appropriate secondary antibody conjugated with HRP at room temperature and washed again. The membrane was then developed using ECL chemiluminescence (Thermo Scientific, Rockford, IL) in a BioRad ChemiDoc™ XRS+ System. β-actin/GAPDH was used as loading control and band intensities were quantified using ImageJ software.

### Gelatin zymography for MMP activity assay

Gelatin zymography was performed using 1.5% gelatin gel as described previously with slight modification [4]. Briefly, kidney cortical tissue was cut into small pieces in ice-cold extraction buffer containing 10 mM cacodylic acid, 20 mM ZnCl, 1.5 mM NaN_3_, and 0.01% Triton X-100 (pH 5.0). The mixture was incubated overnight at 4°C with gentle shaking. Samples were centrifuged at 800 g for 10 min, supernatant collected and protein concentration was measured. Equal amount of protein was separated by 10% SDS-PAGE containing gelatin. After two washes with 2.5% Triton X-100 for 30 min, the gel was rinsed in distilled water twice and incubated in an activation buffer (50 mM Tris-HCl, 5 mM CaCl_2_, and 0.02% NaN_3_, pH 7.5) in a humidified chamber at 37°C for 48 h with gentle shaking. The gel was stained with 0.5% Coomassie solution (acetic acid: methanol: water, 10∶50∶40; v/v) for 1 h. After further wash with distilled water, MMP activity was visualized as white bands against a blue background.

### Statistical Analysis

Results are expressed as mean ± SEM of ‘n’ number of animals in each group. The differences between mean values were analyzed by one-way analysis of variance (ANOVA) using Primer of Biostatistics (version 7). Comparison between groups was made using post hoc Bonferroni correction. Non-parametric data was analyzed using Kruskal-Wallis test followed by Mann-Whitney Rank Sum test. Significance was accepted at p<0.05.

## Results

### Food and water intake

Ang II treated mice consumed less water and food compared to vehicle infused animals (Table 2). A similar trend was noticed in Ang II mice supplemented with FA. There was no significant change in food intake between Ang II + FA and FA alone. However, water intake was significantly higher in FA group compared to Ang II + FA (Table 2).

**Table 2:** Food and water intake.

	Vehicle (n = 4)	Ang II (n = 4)	Ang II + FA (n = 4)	FA (n = 4)
Food intake (gm/day)	5.25±0.38	3.88±0.11*	4.44±0.1*	4.56±0.1
Water intake (mL/day)	8.80±0.49	7.39±0.35*	7.85±0.4*	9.31±0.24^†^

Food and water intake was measured for 5 days. Vehicle group represents animals treated with normal saline; Ang II, angiotensin II; FA, folic acid. Data is presented as mean ± SEM. * p<0.01 vs. vehicle control; ^†^ p<0.05 vs. Ang II + FA.

### Folic acid reduces high blood pressure and plasma Hcy levels

**Figure 1:**
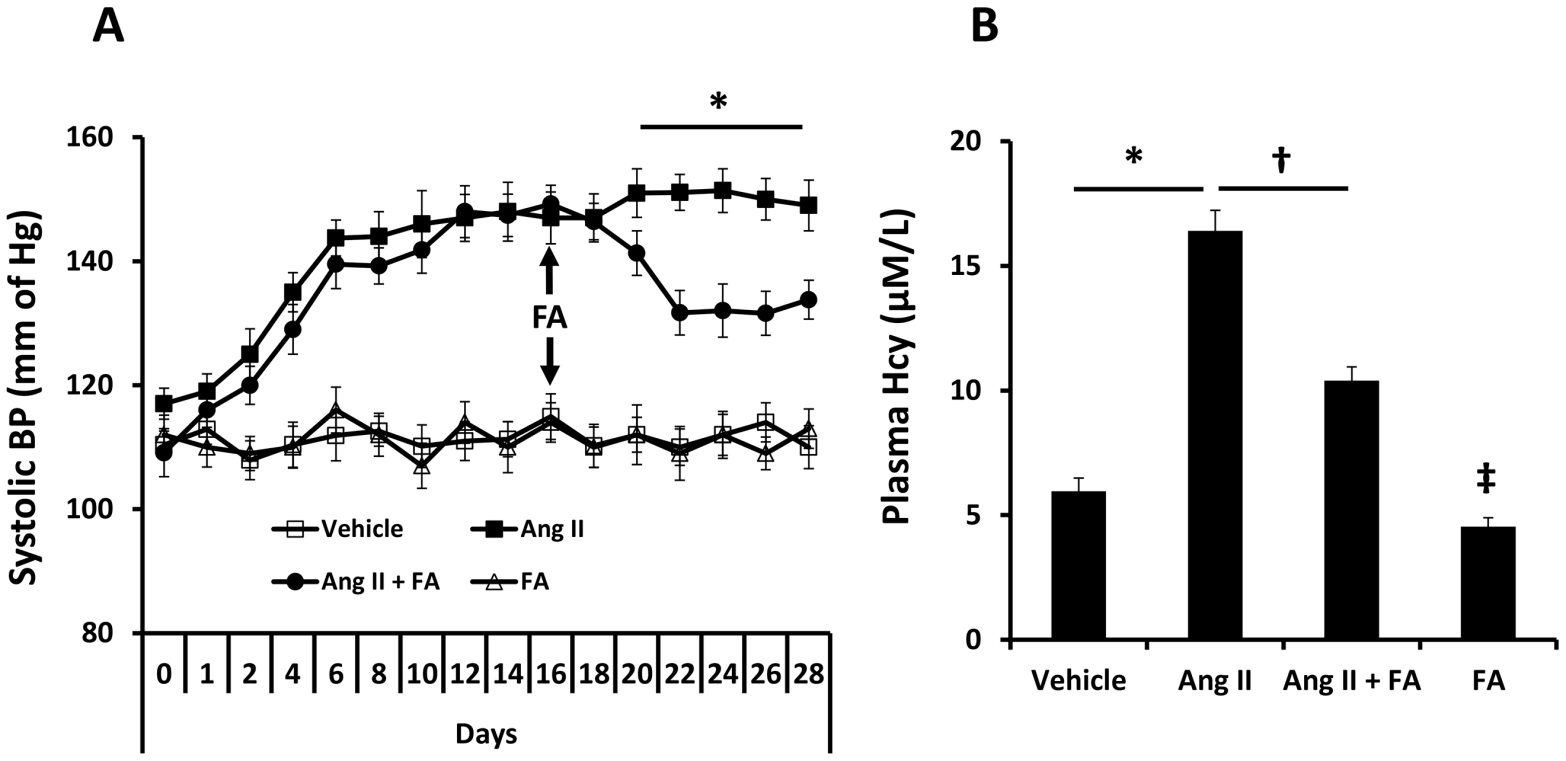
Folic acid (FA) reduced blood pressure and plasma Hcy levels in Ang II-induced hypertension. (**A**) Ang II was infused using alzet mini pump (1000 ng/kg/min) for 4 weeks and blood pressure was measured by radiotelemetry as described in the Materials and Methods. Folic acid (0.015 g/L) was given in drinking water 14 days after pump insertion and continued till the end of the experiment; n = 8 animals/group. * p<0.05 between Ang II + FA vs. Ang II. (**B**) Plasma Hcy was measured by high performance liquid chromatography (HPLC) as described in the Materials and Methods. Data was first analyzed with ANOVA and pairwise comparison was performed using Bonferroni method. Data is presented as mean ± SEM, n = 6 mice/group. * p<0.05 vs. vehicle (saline) and, ^†^ p<0.05 vs. Ang II (1000 ng/kg/min), ^‡^ p = 0.05 vs. Vehicle.

Ang II induced high systolic blood pressure which reached ∼156 mm of Hg within 2 weeks of intraperitoneal infusion (Fig. 1A) and remained constant later till the end of the experiment. Interestingly, FA lowered Ang II-induced high blood pressure gradually reaching a low level one week after commencing treatment and plateaued thereafter. FA alone did not have any effect on blood pressure (Fig. 1A).

We measured plasma homocysteine (Hcy) levels to determine whether increased blood pressure in Ang II infused mice had an effect on their levels. Plasma Hcy levels were increased by almost 3-fold in Ang II infused mice compared to vehicle control (Fig. 1B). FA treatment reduced plasma Hcy significantly in Ang II mice. A marginal reduction in Hcy level was also seen in animals treated with FA alone compared with vehicle control (Fig.1B).

### Renal cortical blood flow and vascular density is improved in Ang II hypertension with FA supplementation

**Figure 2:**
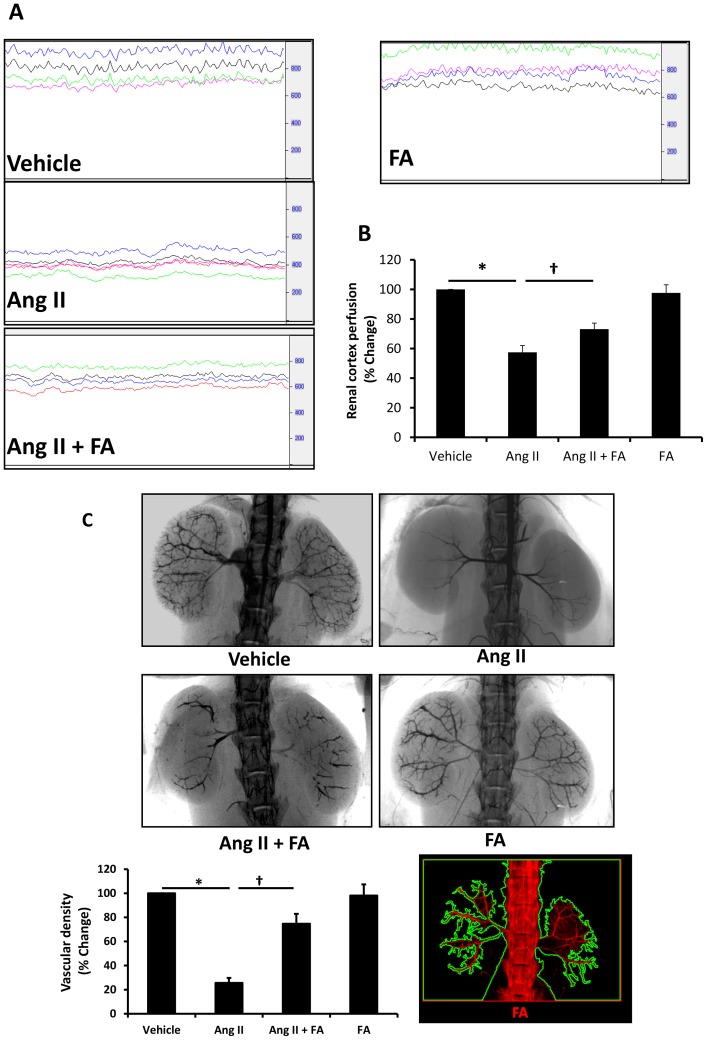
Folic acid treatment increased renal cortical blood flow and vascular density in Ang II infused kidney. (**A**) Renal cortical blood flow was measured at end-point using Speckle contrast Imager (MoorFLPI, Wilmington, DE). Animals were anesthetized with TBE (Tribromoethanol, 240 mg/kg b.w. i.p.) and the left kidney exposed. All measurements were done under standard conditions of light and temperature control. (**B**) Data was first analyzed with ANOVA and pairwise comparison was performed using Bonferroni method. Summarized bar diagram represents mean ± SEM, n = 5–6 animals/group. * p<0.05 vs. vehicle and ^†^p<0.05 vs. Ang II. (**C**) Mice were infused with Barium sulfate (100 mg/ml, at pH 5.0) through PE10 catheter (ID -0.28 MM, Franklin Lakes, NJ) inserted in the carotid artery directed towards the aorta and a constant rate of 200 µL/min was injected. Two minute X-ray images were captured with Kodak 4000 MM image station (Molecular Imaging System; Carestream Health Inc., Rochester, NY). Image analyses were done by ImagePro software (a representative analysis image is shown at the bottom right). Statistical analyses were performed with Kruskal-Wallis test and individual pairs were compared using Mann-Whitney Rank sum test. Bar diagram indicates percent change of vascular density against the background using vehicle treatment as control, n = 3 mice/group. * p<0.05 vs. vehicle; ^†^ p<0.05 vs. Ang II.

Ang II is a potent vasoconstrictor which reduces renal blood flow and homocysteine is known to cause endothelial cell damage and dysfunction. In the present study, we measured changes in the renal cortical blood flow due to Ang II and its associated plasma homocysteine elevation. We also determined whether FA treatment could improve regional blood flow by lowering homocysteine levels. Our results showed that Ang II significantly reduced blood flow in the renal cortex compared to vehicle treated animals (Fig. 2A & B). Interestingly, when Ang II infused animals were supplemented with FA, blood flow improved significantly compared to Ang II treated animals. Blood flow in FA alone group remained constant at baseline level comparable to vehicle control (Fig. 2A & B).

We next determined whether the reduction in renal cortical blood flow was associated with changes in the renal vasculature by soft tissue Barium sulfate angiography. In the Ang II treated animals, there was poor penetration of Barium sulfate in the segmental, arcuate and interlobular arteries suggesting significant blockage (Fig. 2C). Upon FA supplementation, there was restoration of vessel patency in the cortical areas of Ang II treated mice compared to Ang II mice which did not receive FA. Virtually, no change was observed in the vehicle and FA alone treatment groups (Fig. 2C).

### Nitric oxide production is improved with FA treatment in Ang II hypertension

**Figure 3:**
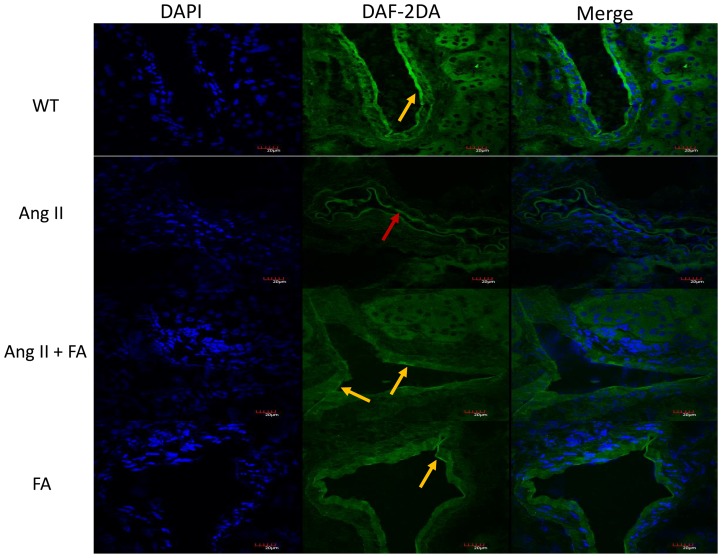
Nitric oxide production is increased with Folic acid treatment in Ang II hypertension. Representative images of DAF-2DA staining to visualize nitric oxide in kidney section. WT animals show normal fluorescence in the inner layer of intrarenal vessels (yellow arrow) whereas, Ang II treated animals show significantly decreased fluorescence (red arrow). NO was increased following FA treatment.

The production of NO is vital to maintaining vascular homeostasis and its reduction is an early sign of endothelial dysfunction. To detect NO formation, we stained tissues with DAF-2DA. There was no difference in the fluorescent intensities (yellow arrows) in the renal vasculature of vehicle and FA treated animals (Fig. 3). A weak fluorescent signal was detected in the inner layer of intrarenal vessels (red arrow) of Ang II treated mice (Fig. 3). The signal intensity increased following FA treatment in Ang II + FA group (yellow arrow). The surrounding kidney tissue did not reveal any change in the DAF-2DA fluorescence in all the groups.

### Nox-2, -4 and superoxide production in the kidney tissue

**Figure 4:**
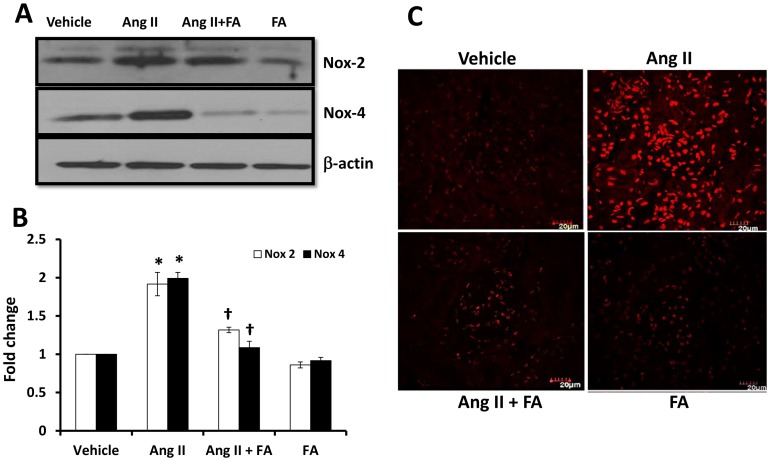
FA treatment reduces ROS production in Ang II hypertension by decreasing Nox-2 and Nox-4 isoforms. (**A**) Equal amount of protein from each group was separated on a SDS-PAGE and immunoblotted with anti-Nox-2, and -4 antibodies. (**B**) The pixel densities of bands from n = 6/group were quantified using ImageJ software (National Institute of Health, NIH) and presented as fold change using β-actin as control. Statistical analyses were performed with Kruskal-Wallis test and individual pairs were compared using Mann-Whitney Rank sum test. * p<0.05 vs. vehicle; p<0.05 vs. Ang II. (**C**) Superoxide was detected in the glomerulus by dihydroethidium (DHE) staining as described in the Materials and Methods. Scale 20 µm.

Reactive oxygen species causes renal and vascular damage by several mechanisms which include inflammation, activation of MMPs and deposition of ECM proteins. We measured NADPH oxidase subunits, Nox-2, and -4 and superoxide production in the tissues to denote the oxidant status. Our results showed that Nox-2 and -4 were upregulated in Ang II hypertension (Fig 4A & B) and FA supplementation mitigated both their expressions (Figs. 4A & B). Corroborating with Nox-2 and -4 expressions, DHE stain revealed markedly increased superoxide production in the glomeruli of Ang II treated animals (Fig. 4C). A reduction in superoxide levels was seen following FA treatment in Ang II hypertensive mice (Fig. 4C). Vehicle treated animals had very low amount of superoxide anions, which remained unchanged in the FA treatment group (Fig. 4C).

### mRNA and protein levels of CBS/CSE/MTHFR is decreased in Ang II hypertension

**Figure 5:**
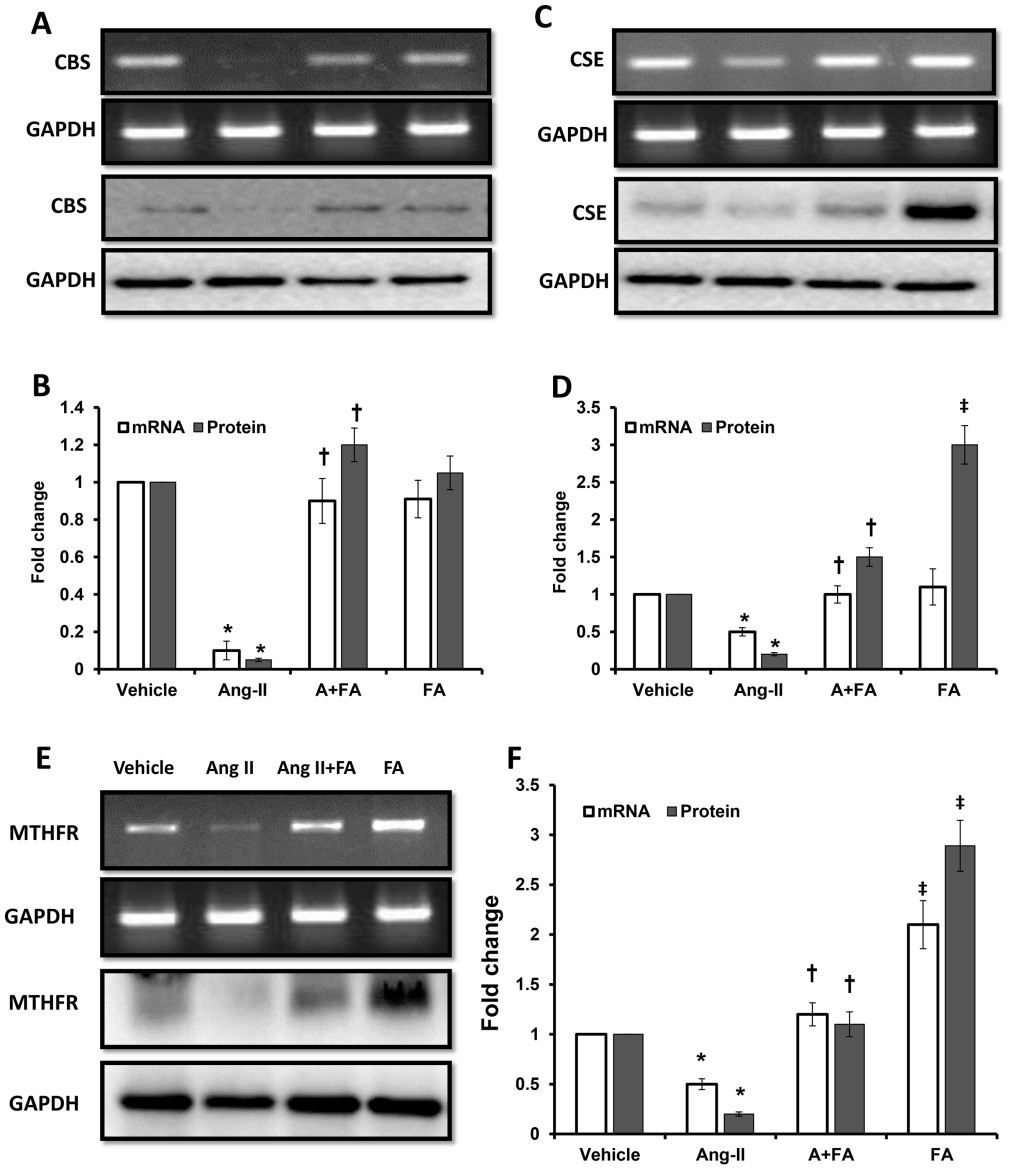
mRNA and protein expression of CBS/CSE and MTHFR is decreased in Ang II induced hypertension Effect of vehicle, Ang II and FA on the mRNA and protein expression of CBS (**A**); CSE (**C**) and MTHFR (**E**) as determined by semiquantitative RT-PCR and Western blotting. Statistical analyses were performed with Kruskal-Wallis test and individual pairs were compared using Mann-Whitney Rank sum test. Bar diagrams represent fold change from n = 6 experiments using GAPDH as control. * p<0.05 vs. vehicle; ^†^ p<0.05 vs. Ang II, ^‡^ p<0.05 vs. vehicle.

Since hypertension is associated with high levels of Homocysteine, we wanted to determine whether this was secondary to altered Hcy metabolism. RT-PCR analysis of renal cortical tissues revealed non-existent cystathionine beta synthase (CBS) mRNA expression in Ang II treated animals (Fig. 5A and B), and cystathionine gamma lyase (CSE) mRNA expression was significantly diminished (Fig. 5C and D). The mRNA expression of methylenetetrahydrofolate reductase (MTHFR) was similarly decreased in Ang II animals (Fig. 5E and F). Corroborating with the above, protein levels of CBS, CSE and MTHFR were decreased significantly in Ang II treated animals (Fig. 5A–F). Following FA supplementation, both the mRNA expression and protein levels of CBS, CSE and MTHFR increased in the Ang II + FA group. FA treatment alone showed a greater increase of protein expression for CSE and MTHFR than CBS (Fig. 5C–F).

### HHcy increases assymetric dimethylarginine (ADMA) and decreases endothelial nitric oxide synthase (eNOS) expression

**Figure 6:**
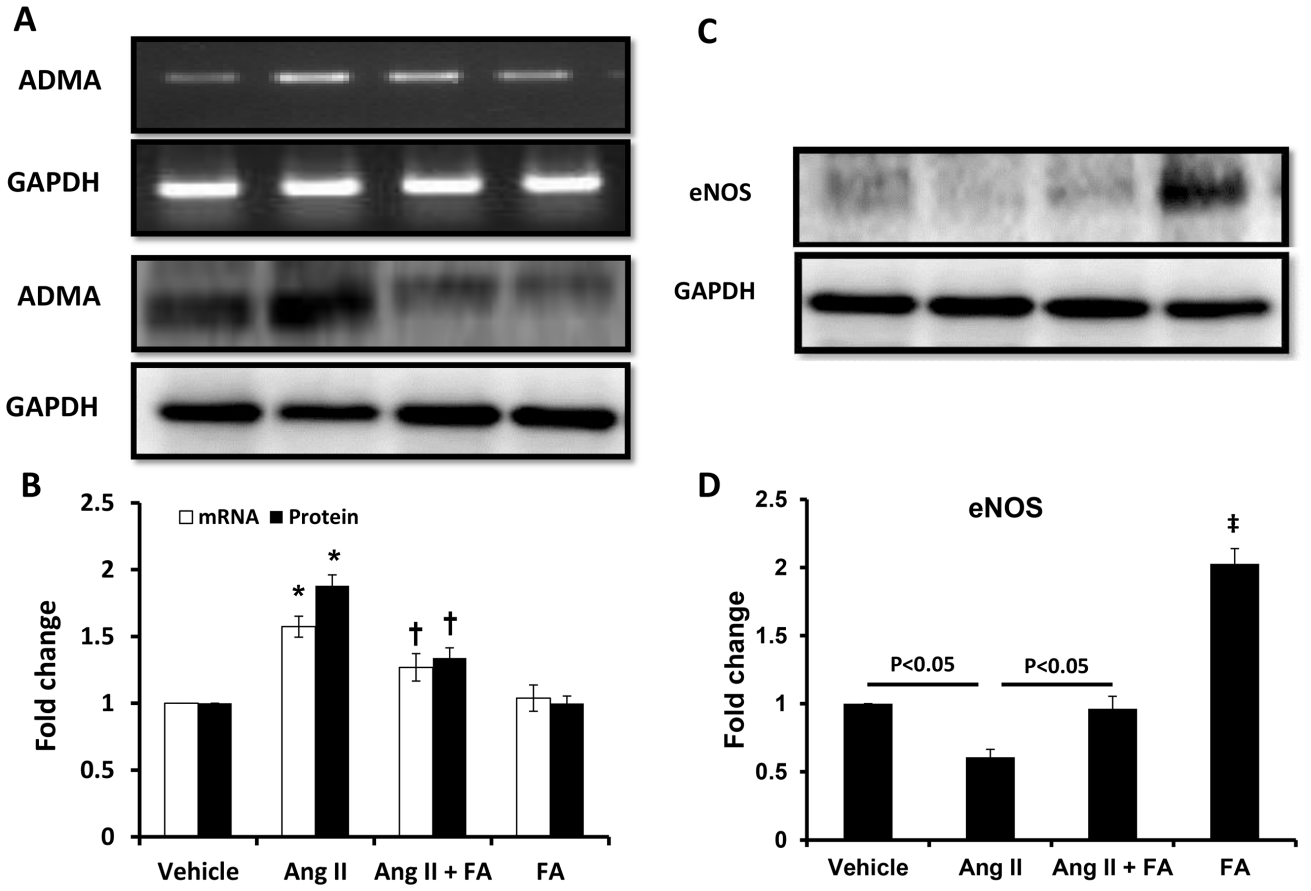
HHcy in Ang II hypertension increases expression of ADMA and suppresses eNOS. Effect of vehicle, Ang II and FA on mRNA and protein expression of ADMA (**A**) and protein expression of eNOS (**C**) by RT-PCR and Western blotting. Statistical analyses were performed with Kruskal-Wallis test and individual pairs were compared using Mann-Whitney Rank sum test. Bar diagrams represent fold change for ADMA (**B**) and eNOS (**C**) from n = 6 animals/group. GAPDH was used as control. * p<0.05 vs. vehicle; ^†^ p<0.05 vs. Ang II, ^‡^ p<0.05 vs. vehicle.

ADMA is an endogenous inhibitor of nitric oxide synthase (NOS), synthesized by methylation of L-arginine by protein arginine methyltransferases. Stuhlinger et al, have demonstrated that Hcy causes ADMA accumulation by inhibiting dimethylarginine dimethylaminohydrolase (DDAH) [29]. We wanted to determine whether HHcy contributed to Ang II hypertension via ADMA inhibition of NO bioavailability. In Ang II treated mice, we found mRNA expression of ADMA was significantly increased and the protein expression was also increased by nearly 1.8 fold (Fig 6A and B). The eNOS protein expression was decreased in Ang II hypertension (Fig. 6C). Following FA treatment, mRNA expression and protein levels of ADMA decreased, and the protein expression of eNOS increased significantly (Fig. 6A–D). Interestingly, FA alone caused a marked increase in the eNOS protein expression (Fig. 6C and D).

### Ang II hypertension decreases angiogenic factor and increases anti-angiogenic factors

**Figure 7:**
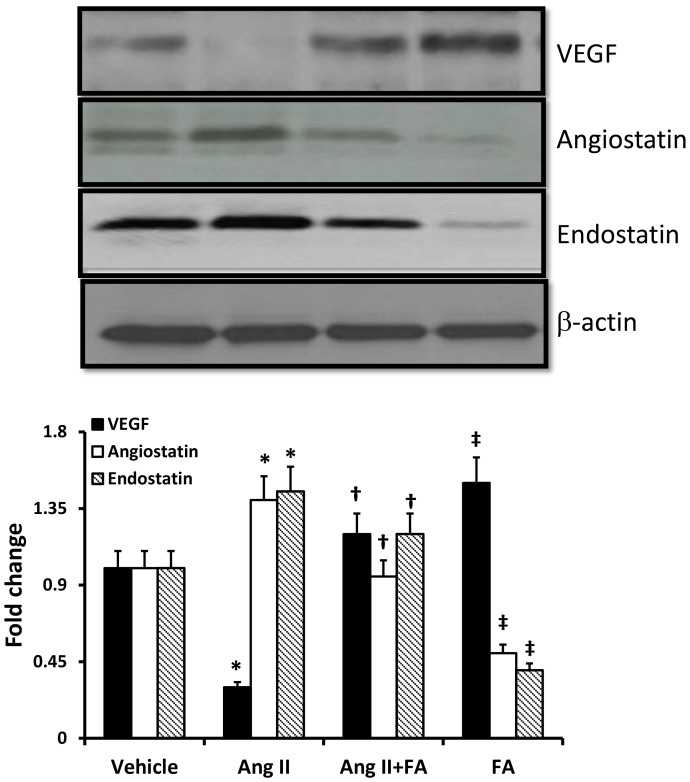
FA normalized altered expression of angiogenic and anti-angiogenic factors in Ang II hypertension. Western blot was performed to measure VEGF, angiostatin and endostatin expression using specific antibodies in the renal cortical tissue extracted protein. Statistical analyses were performed with Kruskal-Wallis test and individual pairs were compared using Mann-Whitney Rank sum test. Bar diagrams represent fold change from n = 5–6 mice/group. * p<0.05 vs. vehicle and ^†^ p<0.05 vs. Ang II, ^‡^ p<0.05 vs. vehicle.

The expression of angiogenic factor, VEGF, was reduced in renal cortical tissue of Ang II-treated mice; whereas, anti-angiogenic factors, angiostatin and endostatin, were up-regulated (Fig. 7). FA treatment ameliorated VEGF, and mitigated angiostatin and endostatin in Ang II-hypertensive mice (Fig. 7).

### Collagen deposition in the kidney

**Figure 8:**
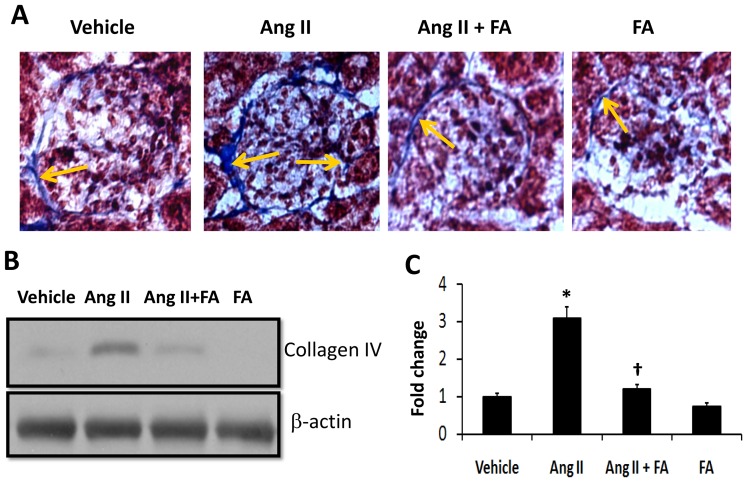
FA mitigated glomerulosclerosis and collagen IV expression in Ang II hypertension. (**A**) Histological kidney sections (5 µm thick) were stained with Masson's trichrome. Collagen is shown as deep blue color (yellow arrows); 200× magnification. (**B**) Collagen IV was detected in the cortical tissue extracted protein by Western blot. (**C**) Bar diagram represents densitometric analysis of collagen IV expression as fold change against vehicle group, n = 5–6 group. Statistical analyses were performed by Kruskal-Wallis test and individual pairs were compared using Mann-Whitney Rank sum test. * p<0.05 vs. vehicle and, ^†^ p<0.05 vs. Ang II.

Renal fibrosis was quantified by Masson's trichrome staining of kidney sections which showed increased collagen deposition in the glomerular and peri-glomerular space (Fig. 8A, arrows). Similarly, immunoblotting of collagen IV in the renal cortical tissue extracted protein showed higher collagen IV expression (Fig. 8B & C). Supplementation of FA reduced collagen deposition in the glomerular and peri-glomerular spaces (Fig. 8A), and collagen IV expression (Fig. 8B & C).

### Alteration of MMPs/TIMPs level and MMP activity in Ang II hypertension

**Figure 9:**
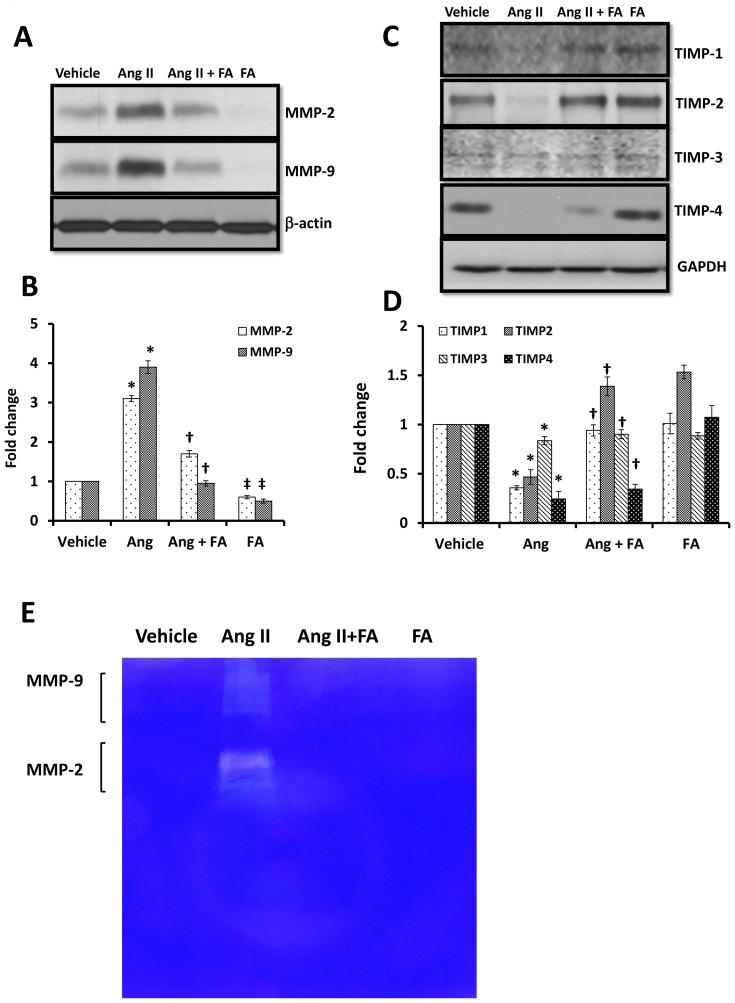
FA normalized MMP/TIMP axis and MMP-2, -9 activities in Ang II hypertension. (**A**) MMP and TIMP expressions were measured in the protein extracted from the renal cortex by Western blot. (**B**) Bar diagram represents densitometric analysis of MMP/TIMP and compared as fold change using vehicle group as control, n = 5-6/group. Statistical analysis was performed with Kruskal-Wallis test followed by Mann-Whitney Rank sum test for individual comparison. * p = 0.02 vs. vehicle and ^†^ p<0.05 vs. Ang II, ^‡^ p<0.05 vs. vehicle. (**C**) In gel gelatin zymography was performed to measure MMP activities of renal cortical tissue extracted protein following our previously adopted method and as described in the Materials and Methods. MMPs activity appeared as light bands against blue background.

Immunodetection of renal cortical tissue extracted protein revealed that in Ang II induced hypertension, the expression of MMP-2 and -9 were upregulated (Figs. 9A & B) whereas, their inhibitory molecules TIMP-2 and -4 were diminished (Fig. 9C & D). TIMP-1 expression was decreased in Ang II treated animals compared to the other groups (Fig 9C & D). Supplementation of FA normalized the expression of MMP-2, - 9 and TIMP-1, -2, but not TIMP-4 (Fig. 9A – D). TIMP-3 expression remained similar in all the groups (Fig. 9C & D). TIMP-4 expression was marginally increased with FA supplementation in Ang II mice (Fig. 9C & D). Although FA did not have any effect on baseline TIMP-2 and -4 protein expression, the expression of MMP-2 and -9 were completely abolished (Fig. 9A).

Since the proteinase activity of MMP-2 and -9 are major determinants of matrix turnover, we measured their activities. Our results showed no detectable activity for MMP-2 and -9 in vehicle treated cortical kidneys (Fig. 9E), however, there was a significant increase in their activities in Ang II treated hypertensive mice (Fig. 9E). FA treatment to Ang II mice mitigated both MMP-2 and -9 activities completely to values similar to vehicle treated mice. FA treatment alone had no effect on both MMP-2 and -9 activities (Fig. 9E).

## Discussion

**Figure 10:**
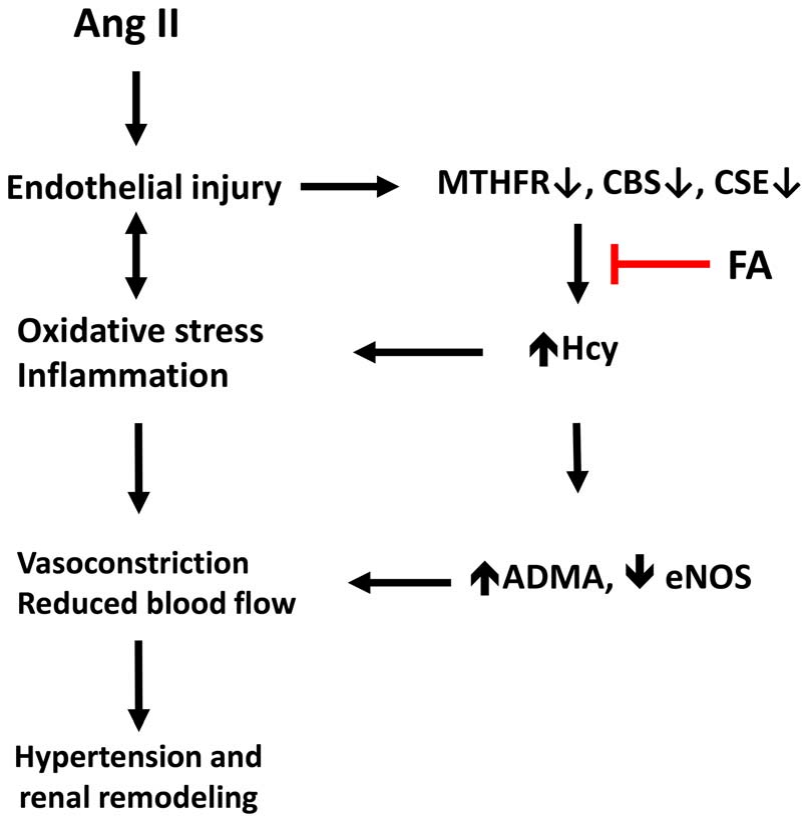
Schematic presentation and possible mechanism of HHcy contribution to hypertension and renal remodeling in Ang II induced hypertension.

Our study suggests that Ang II hypertension causes elevation in plasma homocysteine (Hcy) levels aggravating blood pressure and renovascular remodeling. HHcy in Ang II hypertension occurs as a result of impaired remethylation and transulfuration processes due to decrease in MTHFR and CBS/CSE enzymes respectively. Endothelial dysfunction results from Hcy mediated ADMA accumulation causing NOS inhibition and decreased NO production. Remodeling was characterized by a significant reduction in cortical blood flow, vascular density and glomerular and interstitial fibrosis. The expression of VEGF was attenuated whereas, anti-angiogenic factors, endostatin and angiostatin, were elevated in Ang II hypertension. Interestingly, folic acid (FA) treatment normalized plasma Hcy levels in Ang II mice, and partially mitigated high blood pressure. These changes were associated with an increase in vascular density and normalization of renal cortical blood flow. In addition, FA supplementation mitigated the effect of ADMA on NO synthesis and also increased VEGF expression and reduced anti-angiogenic factors endostatin and angiostatin. A schematic representation of the possible mechanism of HHcy contribution to hypertension and renal remodeling in Ang II induced hypertension is presented in Fig. 10.

The association of hyperhomocysteinemia and high blood pressure has been documented in several clinical studies [1], [30], [31]. However, a similar link between hypertension and homocystinuria has not been reported widely except in isolated cases [32], [33] possibly due to other co-existing pathologies. In a recent study involving HHcy children, supplementation of FA was found to reduce Hcy levels as well as high blood pressure suggesting a causal role for HHcy [34]. A strong correlation has also been observed between elevated plasma Hcy levels and systolic blood pressure which enhanced further if the subjects were also smokers [35].

Folic acid (FA) has numerous roles in health and disease. FA supplementation during preconception period prevents the development of congenital malformations [36]. It was also shown to reduce the risk of stroke in primary prevention [37]. In cancer development, FA has a dual role. During early stages and when the folate levels are low in the body it offers protection; whereas, in advanced stage or at high levels in the body it can accelerate the cancer process. FA has also been reported to reduce the incidence of breast cancer [38] alcohol-associated hepatic cancer [39] and age-related macular degeneration [40]. The above beneficial effects are independent of its role as Hcy reducing agent.

In other studies, FA acid supplementation has been reported to improve endothelial dysfunction in hyperhomocysteinemia (HHcy) patients with vascular disease [41], [42] including asymptomatic HHcy subjects [43], [44]. Interestingly, this beneficial effect was observed even in normal subjects without Hcy elevation [45], [46]. In addition, FA has also been shown to reduce superoxide production [47], endothelial nitric oxide synthase uncoupling, and may also increase purine synthesis and thus sustain ATP levels in the ischemic heart [48]. More recently, in a study by Xun et al involving young adults, higher folate intake was associated with lower incidence of hypertension development in later life [49]. However, its precise mechanism was unknown. In the present study, we show that in Ang II hypertension, increase in blood pressure and subsequent remodeling is partly dependent on the elevation of Hcy levels and FA treatment reduces these effects by remethylating Hcy.

Angiotensin II has multiple effects on the kidney. In addition to vasoconstriction of afferent and efferent arterioles [50] its effect on prostaglandins and mesangial cells can have a variable final result. For example, in mice, Ang II induced activation of prostaglandin EP3 receptor(s) was found to elevate blood pressure [51] and the lack of microsomal prostaglandin E synthase-1 and thus prostaglandin E2 worsened cardiac function following Ang II infusion [52]. Also, Ang II mediates mesangial matrix expansion contributing to glomerular injury and dysfunction [53], [54]. However, it is not known whether Ang II has a role in Hcy synthesis or metabolism. Our study demonstrates that Hcy metabolism enzymes, CBS and CSE are greatly diminished in Ang II-induced hypertension. In addition, reduced remethylation due to decreased MTHFR and volume retention occurring in end-stage renal failure further contributes to hyperhomocysteinemia (HHcy) [55]. It is interesting to note that although FA does not have a role in altering transsulfuration enzymes, it facilitates remethylation process; therefore, decreases Hcy levels in hypertension. Previous studies have demonstrated that high levels of Hcy cause accumulation of ADMA by inhibiting the activity of dimethylarginine dimethylaminohydrolase (DDAH), an enzyme responsible for ADMA metabolism [29]. Further, increased ADMA is known to inhibit nitric oxide synthase thus decreasing nitric oxide bioavailability [56]. Consistent with the above, in our study, we found increased levels and expression of ADMA (Fig 6A and B) and decreased eNOS (Fig. 6C and D). A combination of reduction in Hcy metabolizing enzymes and decreased nitric oxide synthesis leads to endothelial injury. FA treatment has been previously shown to reduce plasma arginine and ADMA in a clinical study [57]. The reversal of ADMA and eNOS following FA treatment seen in this study further support the above findings. Finally, whether Hcy modulates prostaglandins or its actions is not known and warrants a separate study to delineate this role.

HHcy has been shown to increase oxidative stress [58]. Oxidative stress and inflammation are central to renal injury and fibrosis in nephropathy of any origin [59], [60]. The major source of ROS production is NAD(P)H oxidase system and two of its isoforms Nox2 and Nox4 are abundantly expressed in the vascular endothelium [61]. Increased ROS causes cellular injury and release of pro-inflammatory chemokines leading to chronic inflammation resulting in excessive accumulation of ECM proteins and renal fibrosis [61]. In the present study, Ang II increased markers for oxidative stress. A reduction in Hcy levels following FA treatment was associated with decreased vascular injury and partial mitigation of blood pressure.

HHcy has been shown to stimulate vascular smooth muscle cell proliferation, and also alter elastic compliance of the vessels [58]. Previous reports, including our own, have demonstrated that Hcy is a potential risk factor for vascular fibrosis and dysfunction [62]–[65]. In a rodent model of diet induced HHcy, Kumagai et al, demonstrated that Hcy alone causes arterial and arteriolar wall thickening, and tubulointerstitial fibrosis in the kidney and FA administration diminished these changes [65]. In the present study, we show that elevation of plasma Hcy in Ang II hypertension causes peri-glomerular and interstitial fibrosis and FA treatment partially mitigates these changes.

Vascular endothelial growth factor (VEGF) plays an important role in renovascular remodeling during hypertension [66]. As a potent mitogen, it promotes endothelial cell migration and vascular growth [67]. The expression and activity of VEGF is inhibited by anti-angiogenic factors, such as angiostatin and endostatin [68]. In the present study, the expression of VEGF, was decreased and the expression of angiostatin and endostatin was increased (Fig. 7). This result contrasts a previously established role of Ang II on VEGF upregulation in general [69]. One possible explanation for this discrepancy could be due to compensatory vs. decompensatory stage of renal remodeling where VEGF is upregulated in the former and downregulated in the latter stage along with an increase in anti-angiogenic factors. A reduction of vascular density seen in the present study is in agreement with the above and suggests impaired vasculogenesis. FA treatment normalized these factors partially suggesting that Hcy was, in part, responsible for some of these effects. In addition, VEGF is a permeability factor which increases cellular permeability [68]. Thus, in our study it is also possible that the diminished VEGF in renal tissue inhibited vascular permeability, and decreased vascular fenestration required for vessel growth; whereas, FA treatment increased VEGF expression (Fig. 7) and normalized tissue vascularity (Fig. 2C). However, the involvement of this mechanism in Ang II hypertension and HHcy associated renal remodeling requires further study.

In a previous study, we reported that Hcy modulates MMP-9 and collagen synthesis through angiotensin II type 1 receptor (AT_1_R) [70]. In addition, Ang II has been shown to augment vascular collagen deposition [71] and alter compliance [72]. Our finding of increased collagen in the peri-glomerular areas is in concurrence with these earlier reports of Ang II effects (Fig. 10A and B). MMP-2 and -9 have substrate specificity for collagen; therefore, an increase in their activity would reduce collagen accumulation [73]. Contrary to this mechanism, we observed increased collagen deposition in the presence of increased MMP-2 and -9 activities in Ang II hypertension. The possible explanation for this could be: a) increased oxidation of collagen by oxygen radicals and the inability of MMPs to degrade this collagen; b) increased collagen synthesis overwhelming MMP-2 and -9. Indeed, increased collagen turnover has been reported in hypertensive patients [74] further confirming our findings.

HHcy is recognized as an independent risk factor for cardiovascular and neurodegenerative diseases. Genetic mutations in Hcy metabolizing enzymes MTHFR/CBS/Methionine synthase have been described with varying effects on Hcy levels [75]. Mutation of MTHFR gene (C677T) is the most common and has been identified in people with elevated Hcy level [76]. It is unlikely that Ang II can alter or mutate the allele of MTHFR and disrupt the function of this enzyme; however, in the presence of a functional blockage or low levels of co-factors (such as Folate or B_12_ vitamin), Ang II can modify its activity and promote HHcy. Only a MTHFR activity assay can eliminate or endorse this possibility.

Limitations: a) With regard to the reduction in renal cortical blood flow caused by Ang II, we report a large effect; however, some of this difference could be secondary to the anesthetic agent. Since mice are very sensitive to anesthesia and a rapid blood pressure drop can have significant consequences on renal hemodynamics. A better comparison would have been to assess mean arterial pressure simultaneously along with renal blood flow, b) there are several possibilities for Ang II induced homocysteine elevation. One mechanism could involve its metabolism by a methylation process. Since Ang II contains amino acid precursors of catecholamines it is subject to degradation by Catechol-O-methyltransferase (COMT). In Hcy metabolism, S-adenosylmethione is a methyl donor in methyl reactions. Therefore measurement of whether Hcy levels increased first or renal deterioration occurred first could have helped us to understand the cause and effect relationship between Hcy and Ang II.

In conclusion, our study demonstrates that Ang II increases plasma Hcy level and promotes oxidative stress and inflammation. In addition, increased Hcy causes disruption of NO production due to increased accumulation of ADMA and suppression of eNOS suggesting impaired endothelial function and blood flow reduction. Together with an imbalance of MMPs/TIMPs these changes lead to adverse renovascular remodeling with poor functional outcome. A reduction in renal remodeling by FA supplementation suggests that Hcy contributes at least, in part, to the pathophysiological mechanism in Ang II induced hypertension.
